# Twenty-four hour blood pressure variability and the prevalence and the progression of cerebral white matter hyperintensities

**DOI:** 10.1177/0271678X221149937

**Published:** 2023-01-03

**Authors:** Naomi LP Starmans, Frank J Wolters, Annebet E Leeuwis, Esther E Bron, Hans-Peter Brunner La Rocca, Julie Staals, Geert Jan Biessels, L Jaap Kappelle, for the Heart-Brain Connection Consortium

**Affiliations:** 1 Department of Neurology and Neurosurgery, University Medical Centre Utrecht, Utrecht, the Netherlands; 2 Department of Epidemiology, Erasmus MC, Rotterdam, the Netherlands; 3 Department of Radiology & Nuclear Medicine, Erasmus MC, Rotterdam, the Netherlands; 4 Alzheimer Centre Amsterdam, Department of Neurology, Amsterdam Neuroscience, Amsterdam UMC, VU University Medical Centre, Amsterdam, the Netherlands; 5 Department of Cardiology, Maastricht University Medical Centre, Maastricht, the Netherlands; 6 Department of Neurology and School for Cardiovascular Diseases (CARIM), Maastricht University Medical Centre, Maastricht, the Netherlands

**Keywords:** Blood pressure variability, cerebral perfusion, cerebrovascular disease, nocturnal dipping, white matter hyperintensities

## Abstract

Blood pressure variability (BPV) is related to cerebral white matter hyperintensities (WMH), but longitudinal studies assessing WMH progression are scarce. Patients with cardiovascular disease and control participants of the Heart-Brain Connection Study underwent 24-hour ambulatory blood pressure monitoring and repeated brain MRI at baseline and after 2 years. Using linear regression, we determined whether different measures of BPV (standard deviation, coefficient of variation, average real variability (ARV), variability independent of the mean) and nocturnal dipping were associated with WMH and whether this association was mediated or moderated by baseline cerebral perfusion. Among 177 participants (mean age: 65.9 ± 8.1 years, 33.9% female), the absence of diastolic nocturnal dipping was associated with higher WMH volume at baseline (β = 0.208, 95%CI: 0.025–0.392), but not with WMH progression among 91 participants with follow-up imaging. None of the BPV measures were associated with baseline WMH. Only 24-hour diastolic ARV was significantly associated with WMH progression (β = 0.144, 95%CI: 0.030–0.258), most profound in participants with low cerebral perfusion at baseline (p-interaction = 0.042). In conclusion, absent diastolic nocturnal dipping and 24-hour diastolic ARV were associated with higher WMH volume. Whilst requiring replication, these findings suggest that blood pressure patterns and variability may be a target for prevention of small vessel disease.

## Introduction

White matter hyperintensities (WMH) on brain MRI mark the degree of cerebral small vessel disease (SVD) and are associated with an increased risk of stroke and cognitive impairment.^1^ Hypertension is one of the most important risk factors for WMH.^1,2^ Intensive blood pressure lowering therapy slows the progression of WMH by about 40%, as compared with standard blood pressure lowering therapy,^3–5^ but other measures are needed to further reduce WMH accumulation. Increased blood pressure variability (BPV) and an abnormal circadian blood pressure rhythm have emerged as risk factors for stroke and subclinical vascular brain injury.^6,7^

A recent meta-analysis of 27 imaging studies showed that increased 24-hour BPV was associated with an increased burden of SVD on brain MRI.^7^ However, results were heterogeneous and longitudinal studies are scarce, making it difficult to determine causality.^7^ BPV can be expressed through several indices, such as standard deviation (SD) or average real variability (ARV). Reported indices vary considerably across published studies,^7^ and although there is some evidence to suggest ARV may be most suitable for 24-hour BPV,^8^ there are no formal guidelines on preference of one over the other. Arguments in favour of the ARV include its relative robustness to measure BPV irrespective of the physiological decrease in blood pressure that occurs at night as part of the normal circadian rhythm (i.e. nocturnal dipping).^8^ Alterations in the circadian blood pressure rhythm due to autonomic or endocrine dysfunction may lead to the attenuation or absence of the normal physiological nocturnal dip. Measurement of 24-hour BPV can be influenced by changes in these nocturnal blood pressure patterns, and ARV is the most robust of indices to determine BPV irrespective of this nocturnal decrease in blood pressure.^8^

Although BPV overall is associated with more SVD, not all variation in blood pressure is harmful, and nocturnal dipping, as mentioned, is part of the physiological, normal circadian rhythm. Indeed, several cross-sectional studies have shown that the absence of nocturnal dipping is associated with an increased volume of WMH.^9–14^ One population-based longitudinal study in China showed that non-dippers had a significantly higher increase in WMH volume over time,^15^ but whether this translates to non-Asian populations and patients with cardiovascular disease is undetermined.

Despite the link between BPV and WMH, the pathophysiological mechanisms that explain these associations remain uncertain, in particular regarding blood pressure dynamics and the role of cerebral (hypo)perfusion.^16–18^ Sudden decreases in blood pressure with higher BPV could lead to periodic cerebral hypoperfusion and subsequently an increased burden of SVD.^19^ Such changes in blood pressure may be particularly detrimental in patients with already compromised cerebral perfusion. For example, with concomitant obstructive carotid artery disease or heart failure, BPV may put additional strain on the brain and result in more vascular brain injury. Yet, these hypothesis remain to be tested in studies investigating cerebral perfusion in the context of BPV, nocturnal dipping and WMH.

We aimed to determine whether BPV and nocturnal dipping, assessed with 24-hour ambulatory blood pressure monitoring (24-hour ABPM), are associated with the prevalence and progression of WMH volume in a longitudinal study of participants with cardiovascular diseases. We hypothesized that increased BPV and the absence of nocturnal dipping is associated with increased prevalence and progression of WMH volume, and explored the role of cerebral perfusion in these associations.

## Materials and methods

### Study population

This study is part of the Heart-Brain Connection Study, which is a multicentre, prospective, observational study that aims to determine the relation between cardiovascular disease and cognitive impairment, with a focus on hemodynamic factors.^20^ A detailed description of the rationale, inclusion and exclusion criteria has been published previously.^20^ In brief, the cohort is composed of 566 participants aged >50 years, who were diagnosed with vascular cognitive impairment (VCI) (N = 166), carotid occlusive disease (COD) (N = 109) or heart failure (HF) (N = 162). Participants were included between 2014 and 2019 in four participating university medical centres in The Netherlands. We also included 129 control participants. All participants were independent in activities of daily life and able to undergo cognitive testing. Follow-up examination took place after two years.

**Figure 1.**
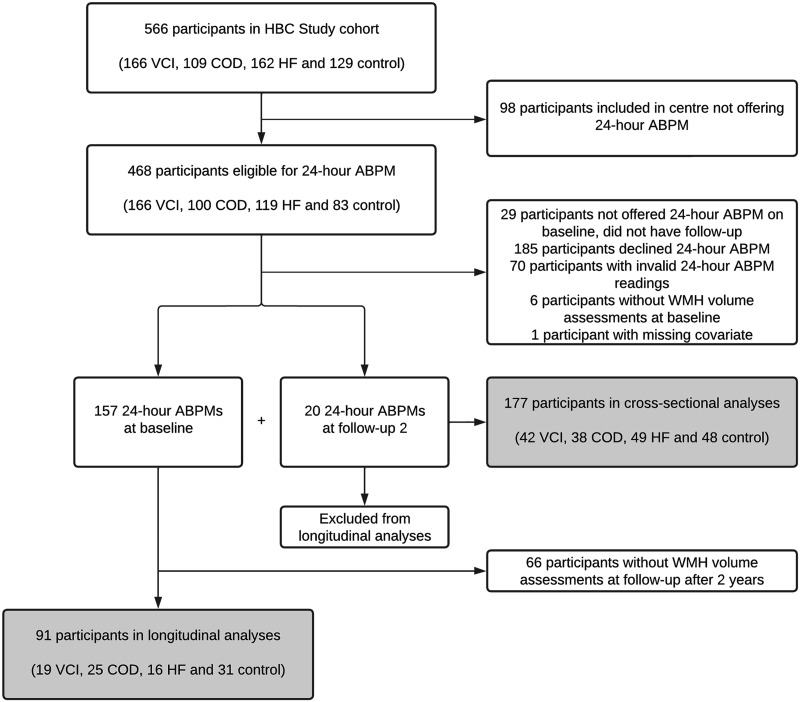
Flowchart of participants inclusion. Routine 24-hour ABPM was incorporated in the Heart-Brain Connection Study protocol after recruitment had already started, resulting in a study sample for the current study that includes 157 of the last 342 consecutively included participants, supplemented by 20 of the earlier included participants who underwent 24-hour ABPM at their two year follow-up visit. ABPM: ambulatory blood pressure measurement; COD: carotid occlusive disease; HBC: Heart-Brain Connection; HF: heart failure; VCI: vascular cognitive impairment; WMH: white matter hyperintensity.

From June 2015 onward, participants were asked to undergo 24-hour ABPM in three out of four participating centres. Patients included prior to June 2015 were invited for ABPM at their two year follow-up visit. For the present study, we included all participants with a valid 24-hour ABPM at either time point (Figure 1). For the cross-sectional analyses, we used the MRI-scan that was performed at the same visit as the 24-hour ABPM.

### Ethics statement

The Heart-Brain Connection Study was approved by the medical ethics committee of Leiden University Medical Centre (P14.002) in agreement with the Declaration of Helsinki. Local medical ethics committees of all sites approved the local performance of the study. All participants provided written informed consent.

### Data availability

Data are available upon reasonable request. All data relevant to the study are included in the article or uploaded as online supplemental information. Requests for access to the data reported in this paper will be considered by the corresponding author.

### Ambulatory blood pressure measurement and blood pressure lowering treatment

Blood pressure was measured non-invasively during 24-hours with validated blood pressure monitors (Microlife WatchBP O3 device, Microlife Corporation, Taiwan).^21^ Measurements were initiated during the visit to the research facility and are thus performed on the same day as the brain MRI. Blood pressure was measured every 20 minutes during daytime (06.00–22.00 hours) and every 60 minutes during nighttime (22.00–06.00 hours). A 24-hour ABPM measurement was considered to be valid, if it had at least 70% valid readings, in line with prior recommendations.^22^

We calculated mean 24-hour systolic (SBP) and diastolic blood pressure (DBP) and different indices of BPV: SD, coefficient of variation (CV), ARV and variability independent of the mean (VIM). We used the following formulas to calculate these indices.^8,23^

$$SD=\;\sqrt{\sum \frac{|BP-{\mathrm{mean}|}^{2}}{n-1}}$$

$$CV=\left(\frac{SD}{\mathrm{mean}}\right)\cdot 100$$

$$\mathrm{ARV}=\;\frac{\sum \left|{BP}_{k+1}-{BP}_{k}\;\right|}{n}$$

$$\mathrm{VIM}=M^{x}\cdot \frac{SD}{{\mathrm{mean}}^{x}}$$

in which *x* can be estimated by $SD=A\cdot {\mathrm{mean}}^{x}$

Of these four indices, ARV has been suggested as the preferred measure to asses 24-hour BPV.^8^ We therefore report ARV among the main results, with other indices presented in the supplementary files. BPV was calculated over 24-hours, as well as stratified by daytime (09.00–21.00 hours) versus nighttime (01.00–06.00 hours). We also calculated the extent of nocturnal dipping by using the following formula: mean nighttime blood pressure/mean daytime blood pressure.

Participants provided a list of their medication, from which we derived the use of blood pressure lowering medication. Administration time of the blood pressure lowering medication was not available. Medication was continued during ABPM. Hypertension was defined as a self-reported history of hypertension or use of blood pressure lowering medication.

### Brain imaging acquisition and processing

In all centres, brain MRI was performed on 3 T scanners (Philips Ingenia, Achieva or Gemini, Philips, Best, The Netherlands) using an eight channel, dual or multi head coil. Participants were scanned on the same scanner and coil during baseline and follow-up examination according to a standardized protocol that included a T1-weighted series (resolution 1 × 1 ×1 mm^3^, magnetization-prepared rapid acquisition gradient echo, repetition time 8.2 ms, echo time 4.5 ms, shot interval 3000 ms, flip angle 8°, inversion delay 990 ms), a fluid-attenuated inversion recovery (FLAIR) sequence (resolution 1.11 × 1.11 × 1.11 mm^3^, repetition time 4800 ms, echo time 313 ms, inversion time 1650 ms, turbo spin-echo factor 182) and 2D phase-contrast flow measurements (resolution 1.17 × 1.17 × 5 mm^3^, repetition time 12 ms, echo time 8.2 ms, flip angle 10°, velocity encoding 200 cm/s, untriggered, 10 averages).^20^ WMH volume was determined by applying an automated brain tissue and WMH segmentation method (Quantib BV, Rotterdam, The Netherlands) to the T1-weighted series and FLAIR images.^24^ WMH segmentations were manually corrected for brain infarcts and volumes were expressed as percentages of total intracranial volume (ICV).

Cerebral blood flow (CBF) was determined with 2D phase-contrast flow measurements in the internal carotid and basilar arteries.^20^ The contours of the basilar artery and both internal carotid arteries were manually drawn using the flow analysis tool of Mass software.^25^ The cross-sectional area of the region of interest was multiplied by the flow velocities in order to obtain the volume flow rates. Total CBF (in ml/min) was then calculated by adding up the volume flow rates through the three vessels. Cerebral perfusion (in ml/min/100 ml) was obtained by dividing the total CBF by the participant’s brain volume and multiplying the result by 100. Cerebral perfusion was then converted into ml/min/100 g by dividing it by the partition coefficient of 0.90.^26^

### Vascular risk factors

Smoking status, either current, former or never, was recorded. Hyperlipidaemia was defined as a self-reported history of hyperlipidaemia or the use of lipid lowering medication. Diabetes mellitus was defined as a self-reported history of diabetes mellitus or the use of anti-diabetic medication. A history of cerebrovascular or cardiovascular disease was defined as a self-reported history of ischemic stroke, transient ischaemic attack, myocardial infarction or peripheral arterial disease. Height and weight were measured during the study visit and were used to calculate the body mass index.

### Statistical analysis

All data were complete with the exception of three participants in whom no data on cerebral perfusion were available. Nocturnal dipping (night/day ratio) was analysed as a continuous variable. Mean 24-hour blood pressure, BPV indices and nocturnal dipping were standardized. WMH volume was expressed as the percentage of ICV and subsequently natural log-transformed to achieve a normal distribution of the data. We assessed normality by visual inspection of Q-Q plots and histograms.

We analysed the association between BPV indices and nocturnal dipping as determinants and the normalized, transformed WMH volume as outcome measure with multiple linear regression, computing both crude estimates and adjusted coefficients with corresponding 95% confidence interval (CI). In the cross-sectional analyses, we adjusted for age, sex, mean 24-hour SBP or DBP (for analyses of SBP or DBP variability, respectively), use of blood pressure lowering medication and body mass index. In the longitudinal analyses, we took the normalized, transformed WMH volume at follow-up as the dependent variable while adjusting for the baseline normalized, transformed WMH volume. In a sensitivity analysis, we defined progression of WMH as an increase in normalized WMH volume exceeding the median increase in the cohort (normalized WMH volume at follow-up – normalized WMH volume at baseline of >0.0180% of ICV) and performed logistic regression to determine if BPV was associated with WMH progression. We also performed a sensitivity analyses in which we excluded participants using beta-blockers as blood pressure lowering medication, because those are associated with the highest 24-hour BPV.^27^

Analyses were performed in the pooled sample of the four participant groups. This is of particular interest because BPV and nocturnal dipping may have a hemodynamic impact, which could be most pronounced in the hemodynamically vulnerable participants of all subgroups. We checked for effect modification by participant group (VCI, COD, HF or control participants) by including interaction terms (BPV or nocturnal dipping * participant group) in the model. In case of significant differences in effects between groups, stratified results were obtained.

Finally, we investigated the role of baseline cerebral perfusion as a potential mediator (calculating direct and indirect effects) or moderator (by adding an interaction term to the model) in the association between BPV or nocturnal dipping and progression of normalized, transformed WMH.

A p-value < 0.05 was considered statistically significant. Analyses were performed in SPSS version 26 and figures were made in R version 4.0.3 (package ggplot2 version 3.3.5). For the mediation analyses, the PROCESS tool for SPSS version 3.5.3 was used.

## Results

Of 254 eligible individuals who underwent 24-hour ABPM, 184 participants had ≥70% valid readings on the ABPM. Of those, we excluded 6 participants with poor quality brain MRI and 1 participant with a missing covariate, leaving 177 participants (69.7%) for analysis (Figure 1 and Supplementary Table 1). Mean age (±SD) was 65.9 ± 8.1 years, 60 (33.9%) were female, and 126 (71.2%) had hypertension (Table 1).

**Table 1.** Baseline characteristics of the participants.

Characteristic	Entire cohortN = 177
Female	60 (33.9)
Age, years	65.9 ± 8.1
Participant group	
Vascular cognitive impairment	42 (23.7)
Carotid occlusive disease	38 (21.5)
Heart failure	49 (27.7)
Control	48 (27.1)
Vascular risk factors	
Current smoking	30 (16.9)
Hypertension	126 (71.2)
Hyperlipidaemia	116 (65.5)
Diabetes mellitus	16 (9.0)
BMI, kg/m^2^	26.7 ± 3.6
History of cerebrovascular or cardiovascular disease	108 (61.0)
Antihypertensive medication	
Any medication	117 (66.1)
Blood pressure measurements	
SBP 24-h mean, mmHg	123.1 ± 14.5
DBP 24-h mean, mmHg	73.7 ± 9.2
SBP 24-h ARV, mmHg	9.8 ± 2.5
DBP 24-h ARV, mmHg	7.8 ± 1.9
SBP nocturnal dipping, ratio	0.89 ± 0.09
DBP nocturnal dipping, ratio	0.86 ± 0.11
WMH volume	
At baseline, ml	1.09 (0.42–5.61)
At baseline, % of ICV	0.08 (0.03–0.41)
At baseline, Fazekas grade 2 or 3	45 (26.5)
At follow-up after 2 years, ml (N = 91)	1.59 (0.52–4.31)
At follow-up after 2 years, % of ICV (N = 91)	0.12 (0.04–0.32)
Delta WMH volume between baseline and follow-up, ml (N = 91)	0.22 (0.00–1.06)
Delta WMH volume between baseline and follow-up, % of ICV (N = 91)	0.02 (0.00–0.08)
Cerebral perfusion	
At baseline, ml/min/100 g (N = 171)	56.1 ± 14.2

Data are presented a N (%) for categorical variables and mean ± SD for continuous variables, except for WMH volume, which is presented as median (interquartile range).

ARV: average real variability; BMI: body mass index; DBP: diastolic blood pressure; ICV: intracranial volume; SBP: systolic blood pressure; WMH: white matter hyperintensity.

BPV increased with age (for 24-hour SBP ARV: r = 0.202, p = 0.007), but did not correlate significantly with nocturnal dipping (for 24-hour SBP ARV and SBP nocturnal dipping: r = −0.075, p = 0.323). BPV was not significantly different between participants with and without hypertension (10.0 ± 2.5 vs. 9.4 ± 2.6 mmHg, p = 0.195). Median WMH volume was 1.09 (IQR 0.42–5.61) ml, corresponding with 0.08 (IQR 0.03–0.41) % of the ICV. Baseline characteristics per participant group (i.e. VCI, COD, HF and control participants) are shown in Supplementary Table 2.

### WMH volume at baseline

The mean 24-hour SBP and DBP were significantly associated with WMH volume at baseline after accounting for potential confounders (Table 2). Twenty-four hour and daytime systolic and diastolic ARV and nocturnal dipping were significantly associated with WMH volume at baseline in the univariable models (Table 2). After adjustment for potential confounders, less DBP nocturnal dipping remained independently associated with a higher WMH volume at baseline (β 0.208, 95% CI 0.025–0.392, p = 0.026). Attenuation of other effect estimates was due mostly to mean 24-hour blood pressure and age. None of the other BPV indices (i.e. SD, CV or VIM) were independently associated with WMH volume at baseline (Supplementary Table 3).

**Table 2.** Cross-sectional associations between blood pressure measurements and WMH volume.

	Crude standardized β(95% CI)	*p*-value	Adjusted standardized β(95% CI)^a^	*p*-value
SBP				
Mean 24-h, mmHg	0.381 (0.172–0.591)	<0.001	0.409 (0.222–0.595)	<0.001
ARV 24-h, mmHg	0.222 (0.008–0.436)	0.042	−0.064 (−0.258–0.130)	0.515
ARV day, mmHg	0.217 (0.002–0.431)	0.047	−0.083 (−0.276–0.110)	0.399
ARV night, mmHg	0.044 (−0.173–0.260)	0.692	−0.008 (−0.192–0.177)	0.935
Nocturnal dipping, ratio	0.300 (0.088–0.512)	0.006	0.164 (−0.022–0.350)	0.084
DBP				
Mean 24-h, mmHg	0.162 (−0.053–0.378)	0.138	0.417 (0.224–0.609)	<0.001
ARV 24-h, mmHg	0.297 (0.085–0.509)	0.006	0.149 (−0.049–0.347)	0.138
ARV day, mmHg	0.303 (0.091–0.515)	0.005	0.125 (−0.070–0.319)	0.207
ARV night, mmHg	0.065 (−0.152–0.281)	0.555	0.093 (−0.093–0.279)	0.323
Nocturnal dipping, ratio	0.289 (0.077–0.502)	0.008	0.208 (0.025–0.392)	0.026

ARV: average real variability; CI: confidence interval; DBP: diastolic blood pressure; SBP: systolic blood pressure; WMH: white matter hyperintensity.

^a^Adjusted for age, sex, mean 24-hour SBP or DBP (for analyses of SBP or DBP determinants respectively), use of blood pressure lowering medication and body mass index.

Associations were broadly similar across participant groups, with the exception of the association of nighttime DBP ARV with WMH, which was different for the patients with VCI compared to the other groups (VCI: β −0.047, 95% CI −0.331–0.236, p = 0.736, other groups: β 0.289, 95% CI 0.056–0.522, p = 0.015).

The sensitivity analyses excluding participants using beta-blockers showed comparable findings (Supplementary Table 4).

### Progression of WMH volume at follow-up

After a median 2.1 years of follow-up (IQR 2.1–2.3), 91 participants underwent repeated MRI. Participants with repeated MRI had a higher mean 24-hour SBP compared with participants who did not have a follow-up visit (Supplementary Table 5). WMH volumes increased on average by 0.22 (IQR 0.00–1.06) ml, corresponding with an increase of 0.02 (IQR 0.00–0.08) % of the ICV. The average increase in WMH volume did not differ significantly between the four participant groups (Supplementary Table 2).

Higher mean 24-hour SBP was significantly associated with increased WMH volume progression after accounting for potential confounders. Twenty-four hour and daytime DBP ARV were significantly associated with progression of WMH volume in the unadjusted model (Table 3). This was broadly unchanged, and remained statistically significant after adjustment for potential confounders (24-hour: β 0.144, 95% CI 0.030–0.258, p = 0.014, daytime: β 0.137, 95% CI 0.026–0.248, p = 0.016). None of the other indices of BPV, nor nocturnal dipping were independently associated with WMH progression (Supplementary Table 6).

**Table 3.** Longitudinal associations between blood pressure measurements and progression of WMH volume (continuous).

	Crude standardized β(95% CI)	*p*-value	Adjusted standardized β(95% CI)^a^	*p*-value
SBP				
Mean 24-h, mmHg	0.111 (−0.007–0.229)	0.064	0.135 (0.018–0.251)	0.024
ARV 24-h, mmHg	0.092 (−0.013–0.196)	0.084	0.028 (−0.083–0.138)	0.617
ARV day, mmHg	0.092 (−0.009–0.193)	0.074	0.024 (−0.082–0.129)	0.657
ARV night, mmHg	0.027 (−0.079–0.134)	0.611	0.013 (−0.091–0.118)	0.802
Nocturnal dipping, ratio	0.020 (−0.104–0.143)	0.752	0.004 (−0.116–0.124)	0.949
DBP				
Mean 24-h, mmHg	0.031 (−0.083–0.144)	0.592	0.092 (−0.024–0.209)	0.119
ARV 24-h, mmHg	0.170 (0.064–0.276)	0.002	0.144 (0.030–0.258)	0.014
ARV day, mmHg	0.175 (0.069–0.281)	0.001	0.137 (0.026–0.248)	0.016
ARV night, mmHg	0.029 (−0.122–0.180)	0.700	0.050 (−0.105–0.206)	0.522
Nocturnal dipping, ratio	−0.063 (−0.183–0.057)	0.302	−0.046 (−0.164–0.071)	0.433

The longitudinal analyses are based on the data of 91 patients. Median follow-up was 2.1 years (IQR 2.1–2.3 years).

ARV: average real variability; CI: confidence interval; DBP: diastolic blood pressure; SBP: systolic blood pressure; WMH: white matter hyperintensity.

^a^Adjusted for age, sex, mean 24-hour SBP or DBP (for analyses of SBP or DBP determinants respectively), use of blood pressure lowering medication and body mass index.

Associations with WMH progression were similar across the different participant groups, except for the association of nighttime SBP ARV with WMH progression. More nighttime SBP ARV related to significantly more WMH progression in the VCI participants, but not in the other groups (VCI: β 0.351, 95% CI 0.069–0.634, p = 0.019, other groups: β −0.054, 95% CI −0.168–0.060, p = 0.347).

In the sensitivity analysis with WMH volume progression as a dichotomous outcome variable, 24-hour and daytime DBP ARV related to a 40% (CI 95% −17 to 136%) and 55% (95% CI −8 to 160%) increased risk of WMH progression, respectively, albeit not statistically significant (Supplementary Table 7). When excluding participants using beta-blockers, the effect estimate of 24-hour DBP ARV somewhat increased (β 0.201, 95% CI 0.066–0.336, p = 0.004), while the other effect estimates remained approximately unchanged (Supplementary Table 8).

### Mediation and effect modification by cerebral perfusion

**Figure 2.**
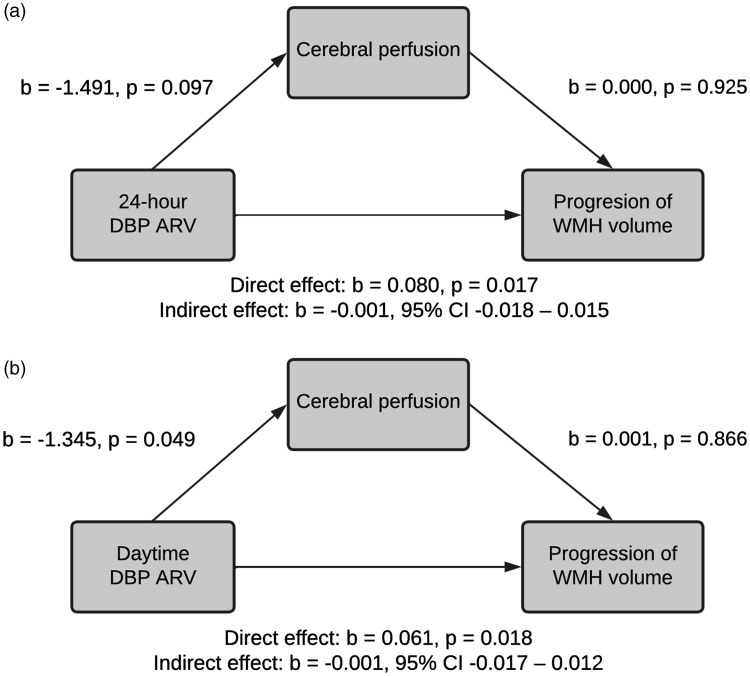
Mediation of the association between BPV and WMH progression by cerebral perfusion, for 24-hour DBP ARV (a), and daytime DBP ARV (b). The indirect effect indicates the effect of BPV on WMH progression through cerebral perfusion. In both panels, mediation is absent due chiefly to lack of association of baseline cerebral perfusion with WMH progression. Models are adjusted for WMH volume at baseline, age, sex, 24-hour mean DBP, use of blood pressure lowering medication and body mass index. ARV: average real variability; BPV; blood pressure variability; DBP: diastolic blood pressure; WMH: white matter hyperintensity.

**Figure 3.**
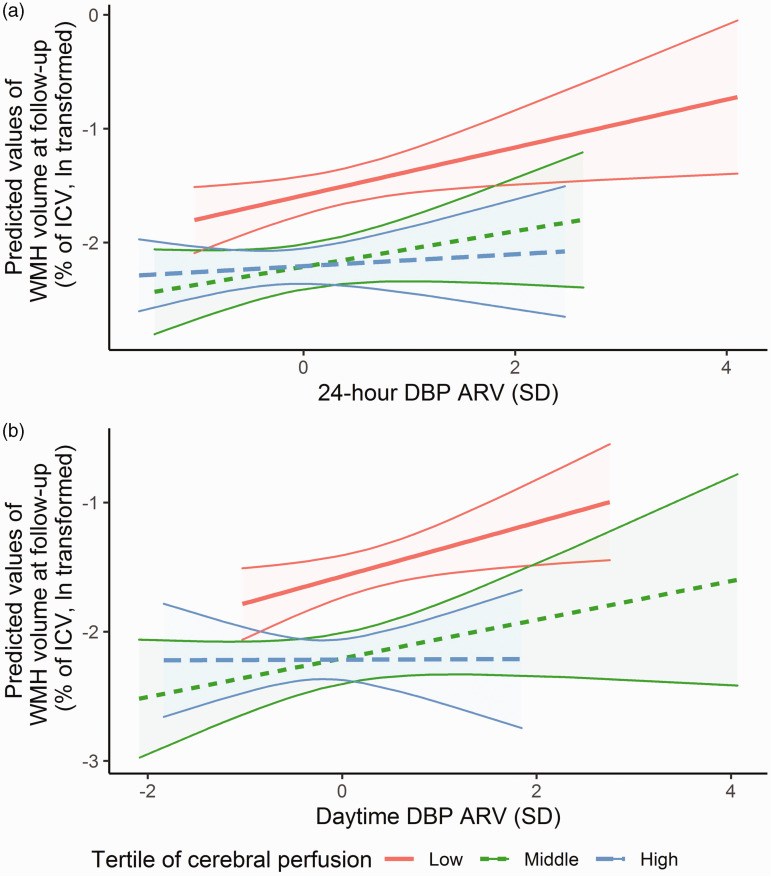
Effect modification of the relation between BPV and progression of WMH by cerebral perfusion, depicted for 24-hour DBP ARV (a), and daytime DBP ARV (b). The association between BPV and progression of WMH volume at follow-up is stratified by baseline levels of cerebral perfusion, and adjusted for age, sex, 24-hour mean DBP, use of blood pressure lowering medication and body mass index. Cut-off values for low perfusion were ≤ 49.4 ml/min/100 g, middle perfusion 49.5–63.0 ml/min/100 g and high perfusion ≥ 63.1 ml/min/100 g. The shading indicates the 95% confidence intervals. ARV: average real variability; BPV: blood pressure variability; DBP: diastolic blood pressure; ICV: intracranial volume; WMH: white matter hyperintensity.

Cerebral perfusion was not associated with progression of WMH over time (Figure 2 and Supplementary Table 9). Consequently, cerebral perfusion did not mediate the association between nocturnal dipping or BPV and WMH volume progression at follow-up. However, associations of 24-hour and daytime DBP ARV with WMH progression were more profound with lower baseline cerebral perfusion (Figure 3 and Supplementary Table 10; p-values for interaction = 0.042 for 24-hour and 0.027 for daytime ARV, respectively). No such differences were observed for nocturnal dipping (Supplementary Table 10).

## Discussion

In this clinical cohort of patients with cardiovascular disease and age-matched control participants, we confirmed the known association between the mean 24-hour blood pressure and the presence and progression of WMH volume. Additionally, we found that less DBP nocturnal dipping was independently related to more WMH at baseline. Higher 24-hour and daytime DBP ARV predisposed to more progression of WMH during two years of follow-up, most profound with low baseline cerebral perfusion. These findings suggest that measurement of BPV and of the circadian blood pressure rhythm may be a target for prevention of SVD.

Of the various studies investigating the association between 24-hour BPV and WMH volume, three studies used ARV as the index of BPV. A retrospective, cross-sectional study of 140 patients with hypertension, but no clinical manifestations of cerebrovascular disease, did not find a difference in 24-hour ARV between patients with none to limited (Fazekas 0–1) versus extensive WMH (Fazekas 2–3).^10^ Another cross-sectional study of 487 patients with hypertension showed that higher 24-hour ARV was significantly associated with a higher prevalence of a composite outcome of either a lacunar infarct or WMH Fazekas grade 2–3, driven by nighttime and SBP variability.^28^ Only one longitudinal study investigated ARV in relation to the progression of WMH or new lacunes during follow-up.^29^ Among 210 community-dwelling individuals aged 70–72 years in Japan, 24-hour SBP and DBP ARV were related to SVD progression, but only in a subgroup of patients who already had SVD at baseline.^29^

Besides ARV, we did not observe associations of any other index of BPV (SD, CV or VIM) with WMH volume at baseline or follow-up. By contrast, two previous cross-sectional studies did find that patients with increased 24-hour systolic SD had a higher burden of SVD.^10,30^ A longitudinal population-based study also found that daytime systolic SD resulted in increased progression of WMH after 5 years of follow-up.^31^ Two studies investigating the CV, one in a population-based cohort^13^ and the other among patients with hypertension,^28^ did not find an association with WMH volume or SVD, whereas one cross-sectional study of relatively healthy participants did report that increased systolic CV was related to more SVD.^30^ Diastolic BPV was assessed in only one cross-sectional study (expressed as SD, CV, and ARV), in which it was unrelated to the presence of SVD in patients with hypertension.^28^ The differences with our results may be explained by differences in study populations. The abovementioned studies often excluded patients with a history of stroke,^10,13,28,30,31^ cognitive impairment,^10,28,30^ a significant stenosis of the internal carotid artery,^10,30^ or severe heart disease.^30,31^ Taken together, BPV seems to play a role in the prevalence and progression of WMH, but depending on the population (healthy participants versus patients with cardiovascular disease) and the type of BPV index used, this role may be larger or smaller.

The heterogeneity in findings between studies could in part be explained by the different BPV indices used. Although the optimal measure remains to be determined,^7^ the resilience of ARV to confounding by nocturnal blood pressure dips is an important advantage of using this metric when assessing 24-hour BPV.^8^ When using SD, CV, or VIM, information bias due to admixture of nocturnal dipping patterns in 24-hour variability may attenuate associations to the null. A head-to-head comparison of the most commonly used BPV indices in the same study population can help to disentangle the properties of the different indices. As such, our findings support the routine use of the ARV for assessing 24-hour BPV.

Regarding nocturnal dipping, our results are in line with a prior meta-analysis of five cross-sectional studies that showed participants with less nocturnal dipping had a higher WMH volume at baseline.^32^ However, in the meta-analysis, this difference was not observed in studies that accounted for potential confounders like age, sex, and traditional cardiovascular risk factors.^32^ So far, only one study has reported on the association between nocturnal dipping and WMH volume longitudinally.^15^ This population-based cohort of Chinese participants without cardiovascular disease, reported that the absence of a nocturnal blood pressure dip was associated with a significantly larger increase in WMH volume during five years of follow-up.^15^ Although, we did not replicate this last finding, administering blood pressure lowering medication at bedtime rather than in the morning, the so called chronotherapy, has been shown to restore the natural circadian rhythm of blood pressure and also to reduce the risk of cardiovascular events, including stroke, in the Hygia Chronotherapy Trial,^33^ but not in the more recent TIME study.^34^ It is currently unknown if chronotherapy reduces the risk of (subclinical) SVD.

Twenty-four hour BPV reflects the central sympathetic drive, autoregulatory mechanisms, and arterial compliance.^8^ Impaired blood pressure regulation can be a consequence of reduced sympathetic drive due to brain injury, but BPV could also contribute to WMH through direct reduction in cerebral perfusion.^16,17,19^ Under normal physiological circumstances, arterial elasticity and compliance help to maintain continuous organ perfusion by dampening fluctuations in blood pressure (i.e. the Windkessel effect).^35^ When arterial compliance is reduced with arteriosclerosis, this may lead to impaired organ perfusion with higher BPV. On the basis of the Windkessel effect, we postulate that changes in perfusion may occur in particular with fluctuations in diastolic rather than systolic blood pressure, which may prompt closer attention for diastolic BPV in future studies. The observed effect modification by baseline cerebral perfusion in our study supports the notion that low perfusion could render participants more vulnerable to large fluctuations in BPV, while participants with relatively high cerebral perfusion may be more resilient. The lack of mediation by cerebral perfusion in our study was due, at least in part, to the absence of an association between baseline cerebral perfusion and WMH. As various prior studies did observe lower cerebral perfusion in individuals with more severe WMH burden,^19^ potential mediation effects by perfusion warrant further assessment in other cohorts. Simultaneous measurement of carotid pulse-wave velocity, intracranial pulsatility, pulse pressure, and cerebrovascular reactivity in such studies may aid to understanding the association between BPV and WMH.^17^

A strength of this study is the longitudinal design, which enabled us to examine WMH progression and shed more light on the role of cerebral perfusion. There are also limitations that should be taken into account. First, the number of participants was relatively small, which hampers precision. Attrition over time may have led - most likely - to an underestimation of true effect estimates due to selection. Second, although we adjusted our analysis for the most important confounders, we could not account for all potential confounding factors. Third, heterogeneity of the study population may hinder interpretability, even though associations were generally similar across participant groups. Fourth, longer follow-up beyond two years is needed to entirely rule out reverse causation. Fifth, a single resting-state measurement of cerebral perfusion is unlikely to fully capture the variability in cerebral perfusion that may be expected to accompany the variability in blood pressure during the day and night. Sixth, BPV is influenced by blood pressure lowering medication, which was continued during 24-hour ABPM. Lastly, 24-hour ABPM was not repeated at follow-up, so we cannot be certain that the BPV observed at baseline persisted for two years.

In conclusion, diastolic nocturnal dipping was associated with WMH volume, and 24-hour diastolic ARV with progression of WMH volume over time, most profound in participants with low cerebral perfusion at baseline. Whilst requiring replication, these findings suggest that blood pressure patterns and variability may be a target for prevention of SVD.

## Supplemental Material

sj-pdf-1-jcb-10.1177_0271678X221149937 - Supplemental material for Twenty-four hour blood pressure variability and the prevalence and the progression of cerebral white matter hyperintensities Click here for additional data file. Supplemental material, sj-pdf-1-jcb-10.1177_0271678X221149937 for Twenty-four hour blood pressure variability and the prevalence and the progression of cerebral white matter hyperintensities by Naomi LP Starmans, Frank J Wolters, Annebet E Leeuwis, Esther E Bron, Hans-Peter Brunner La Rocca, Julie Staals, Geert Jan Biessels, L Jaap Kappelle and for the Heart-Brain Connection Consortium in Journal of Cerebral Blood Flow & Metabolism
