# Supplementary material for: Twenty-four hour blood pressure variability and the prevalence and the progression of cerebral white matter hyperintensities
Source: J Cereb Blood Flow Metab. 2023 Jan 3;43(5):801–11. doi: 10.1177/0271678X221149937 (PMC10108197; doi:10.1177/0271678X221149937)
Supplement: sj-pdf-1-jcb-10.1177_0271678X221149937 - Supplemental material for Twenty-four hour blood pressure variability and the prevalence and the progression of cerebral white matter hyperintensities [file sj-pdf-1-jcb-10.1177_0271678X221149937.pdf]

Supplementary material

Supplementary Table 1. Comparison of baseline characteristics for included and excluded participants.

**Characteristic**	**Included**<br><br>**N = 177**	**ABPM performed, but excluded**<br>**N = 77**	**p-value**
Female	60 (33.9)	29 (37.7)	0.570
Age, years	65.9 ± 8.1	67.2 ± 8.8	0.272
**Participant group**			
Vascular cognitive impairment	42 (23.7)	14 (18.2)	0.411
Carotid occlusive disease	38 (21.5)	29 (37.7)	0.009
Heart failure	49 (27.7)	19 (24.7)	0.647
Control	48 (27.1)	15 (19.5)	0.210
**Vascular risk factors**			
Current smoking	30 (16.9)	21 (27.3)	0.063
Hypertension	126 (71.2)	61 (79.2)	0.216
Hyperlipidaemia	116 (65.5)	60 (77.9)	0.055
Diabetes mellitus	16 (9.0)	21 (27.3)	< 0.001
BMI, kg/m^2	26.7 ± 3.6	27.0 ± 4.8	0.532
History of cerebrovascular or cardiovascular disease	108 (61.0)	54 (70.1)	0.201
**Antihypertensive medication**			
Any medication	117 (66.1)	54 (70.1)	0.563
**WMH volume**	N = 177	N = 70	
At baseline, ml	1.09 (0.42-5.61)	1.23 (0.42 – 4.32)	0.854
At baseline, % of ICV	0.08 (0.03-0.41)	0.09 (0.03 – 0.31)	0.850
At baseline, Fazekas grade 2 or 3	45 (26.5)	22 (32.8)	0.340
**Cerebral perfusion**	N = 171	N = 65	
At baseline, ml/min/100 g	56.1 ± 14.2	53.0 ± 13.9	0.130

Data are presented a N (%) for categorical variables and mean ± SD for continuous variables, except for WMH volume, which is presented as median (interquartile range). The first column is composed of the 177 participants that are included in the cross-sectional analyses of this study. The second column includes the 77 participants that underwent 24-hour ABPM, but were excluded due to an invalid 24-hour ABPM (N = 70), unavailable brain MRI (N = 6) or missing covariate (N = 1).

ABPM: ambulatory blood pressure measurement; BMI: body mass index; ICV: intracranial volume; WMH: white matter hyperintensity.

Supplementary Table 2. Baseline characteristics of the patients by participant group.

Characteristic	VCI<br>N = 42	COD<br>N = 38	HF<br>N = 49	Control<br>N = 48	p-value
Female	17 (40.5)	8 (21.1)	13 (26.5)	22 (45.8)	0.049
Age, years	64.7 ± 7.0	66.8 ± 7.0	67.1 ± 9.9	65.1 ± 7.9	0.371
**Vascular risk factors**					
Current smoking	9 (21.4)	7 (18.4)	9 (18.4)	5 (10.4)	0.548
Hypertension	37 (88.1)	29 (76.3)	47 (95.9)	13 (27.1)	< 0.001
Hyperlipidaemia	32 (76.2)	36 (94.7)	37 (75.5)	11 (22.9)	< 0.001
Diabetes mellitus	4 (9.5)	7 (18.4)	5 (10.2)	0 (0.0)	0.012
BMI, kg/m^2	26.8 ± 3.9	27.2 ± 3.6	26.9 ± 3.8	25.8 ± 3.2	0.319
History of cerebrovascular or cardiovascular disease	32 (76.2)	35 (92.1)	39 (79.6)	2 (4.2)	< 0.001
**Antihypertensive medication**					
Any medication	34 (81.0)	25 (65.8)	47 (95.9)	11 (22.9)	< 0.001
**Blood pressure measurements**					
SBP 24-h mean, mmHg	127.9 ± 16.5	128.3 ± 13.2	115.7 ± 13.2	122.4 ± 11.7	< 0.001
DBP 24-h mean, mmHg	77.4 ± 10.5	73.3 ± 6.6	69.7 ± 9.5	74.8 ± 8.2	0.002
SBP 24-h ARV, mmHg	9.8 ± 2.6	11.1 ± 3.0	9.5 ± 2.3	9.2 ± 2.0	0.017
DBP 24-h ARV, mmHg	8.1 ± 2.3	8.2 ± 1.6	7.7 ± 1.8	7.4 ± 1.7	0.093
SBP nocturnal dipping, ratio	0.91 ± 0.10	0.87 ± 0.09	0.90 ± 0.09	0.87 ± 0.08	0.090
DBP nocturnal dipping, ratio	0.89 ± 0.12	0.84 ± 0.11	0.87 ± 0.11	0.82 ± 0.10	0.055
**WMH volume**					
At baseline, ml	5.78 (1.06-16.66)	0.80 (0.36-1.97)	1.08 (0.41-3.89)	0.70 (0.28-2.19)	< 0.001
At baseline, % of ICV	0.43 (0.08-1.21)	0.06 (0.02-0.14)	0.08 (0.03-0.30)	0.05 (0.02-0.16)	< 0.001
At baseline, Fazekas grade 2 or 3	23 (56.1)	4 (11.4)	10 (21.3)	8 (17.0)	< 0.001
At follow-up after 2 years, ml (N = 91)	3.04 (1.22-13.03)	1.40 (0.41-3.72)	2.39 (1.13-8.84)	1.03 (0.31-2.45)	0.010
At follow-up after 2 years, % of ICV (N = 91)	0.21 (0.09-1.00)	0.08 (0.03-0.25)	0.18 (0.08-0.61)	0.06 (0.03-0.19)	0.011
Delta WMH volume between baseline and follow-up, ml (N = 91)	0.38 (-0.34-1.29)	0.17 (-0.03-1.11)	0.61 (0.00-2.61)	0.21 (0.01-0.63)	0.623
Delta WMH volume between baseline and follow-up, ml, % of ICV (N = 91)	0.03 (-0.02-0.09)	0.01 (-0.00-0.07)	0.04 (0.00-0.18)	0.02 (0.00-0.04)	0.686
**Cerebral perfusion**					
At baseline, ml/min/100 g (N = 171)	59.4 ± 14.9	48.0 ± 13.1	58.5 ± 15.5	57.3 ± 10.7	0.002

Data are presented a N (%) for categorical variables and mean ± SD for continuous variables, except for WMH volume, which is presented as median (interquartile range).
ARV: average real variability; BMI: body mass index; COD: carotid occlusive disease; DBP: diastolic blood pressure; HF: heart failure; ICV: intracranial volume; SBP: systolic blood pressure; VCI: vascular cognitive impairment; WMH: white matter hyperintensity.

Supplementary Table 3. Cross-sectional associations between blood pressure measurements and WMH volume.

		Stratum	Crude standardized β (95% CI)	p-value	Adjusted standardized β (95% CI)[a]	p-value
Systolic blood pressure	**24-hour**					
	Mean, mmHg	-	0.381 (0.172 – 0.591)	< 0.001	0.409 (0.222 – 0.595)	< 0.001
	SD, mmHg	-	0.087 (-0.129 – 0.303)	0.429	-0.084 (-0.278 – 0.110)	0.395
	CV, %	-	-0.078 (-0.294 – 0.139)	0.479	-0.067 (-0.250 – 0.116)	0.472
	ARV, mmHg	-	0.222 (0.008 – 0.436)	0.042	-0.064 (-0.258 – 0.130)	0.515
	VIM, mmHg	-	-0.039 (-0.255 – 0.178)	0.725	-0.069 (-0.250 – 0.113)	0.455
	Nocturnal dipping, ratio	-	0.300 (0.088 – 0.512)	0.006	0.164 (-0.022 – 0.350)	0.084
	**Daytime**					
	SD, mmHg	-	0.264 (0.050 – 0.477)	0.016	0.027 (-0.165 – 0.219)	0.782
	CV, %	-	0.155 (-0.060 – 0.371)	0.157	0.050 (-0.141 – 0.241)	0.608
	ARV, mmHg	-	0.217 (0.002 – 0.431)	0.047	-0.083 (-0.276 – 0.110)	0.399
	VIM, mmHg	-	0.210 (-0.004 – 0.425)	0.055	0.039 (-0.148 – 0.227)	0.679
	**Nighttime**					
	SD, mmHg	-	-0.013 (-0.230 – 0.205)	0.910	-0.104 (-0.287 – 0.079)	0.264
	CV, %	-	-0.152 (-0.368 – 0.065)	0.169	-0.121 (-0.300 – 0.059)	0.186
	ARV, mmHg	-	0.044 (-0.173 – 0.260)	0.692	-0.008 (-0.192 – 0.177)	0.935
	VIM, mmHg	-	-0.096 (-0.313 – 0.121)	0.384	-0.113 (-0.293 – 0.066)	0.214
Diastolic blood pressure	**24-hour**					
	Mean, mmHg	-	0.162 (-0.053 – 0.378)	0.138	0.417 (0.224 – 0.609)	< 0.001
	SD, mmHg	-	0.076 (-0.141 – 0.292)	0.492	0.001 (-0.191 – 0.193)	0.992
	CV, %	-	0.009 (-0.208 – 0.225)	0.938	0.022 (-0.163 – 0.207)	0.814
		VCI	0.214 (-0.259 – 0.687)	0.366	0.102 (-0.339 – 0.543)	0.641
		COD	0.116 (-0.292 – 0.523)	0.569	0.243 (-0.173 – 0.660)	0.243
		HF	0.188 (-0.212 – 0.587)	0.350	0.225 (-0.093 – 0.544)	0.161
		Control	-0.170 (-0.541 – 0.201)	0.361	-0.203 (-0.509 – 0.104)	0.189
	ARV, mmHg	-	0.297 (0.085 – 0.509)	0.006	0.149 (-0.049 – 0.347)	0.138
	VIM, mmHg	-	0.036 (-0.180 – 0.253)	0.741	0.012 (-0.169 – 0.193)	0.895
		VCI	0.204 (-0.247 – 0.655)	0.366	0.092 (-0.330 – 0.514)	0.660
		COD	0.132 (-0.290 – 0.553)	0.531	0.233 (-0.168 – 0.634)	0.245
		HF	0.186 (-0.215 – 0.587)	0.355	0.217 (-0.105 – 0.539)	0.182
		Control	-0.154 (-0.524 – 0.217)	0.408)	-0.208 (-0.508 – 0.091)	0.168
	Nocturnal dipping, ratio	-	0.289 (0.077 – 0.502)	0.008	0.208 (0.025 – 0.392)	0.026

	**Daytime**					
	SD, mmHg	-	0.250 (0.037 – 0.464)	0.022	0.102 (-0.087 – 0.291)	0.287
	CV, %	-	0.229 (0.015 – 0.444)	0.036	0.147 (-0.042 – 0.337)	0.127
		VCI	0.168 (-0.319 – 0.654)	0.490	-0.056 (-0.540 – 0.429)	0.817
		COD	0.441 (-0.069 – 0.950)	0.088	0.638 (0.093 – 1.184)	0.023
		HF	0.255 (-0.115 – 0.624)	0.172	0.265 (-0.028 – 0.559)	0.075
		Control	0.159 (-0.197 – 0.516)	0.373	-0.015 (-0.340 – 0.309)	0.925
	ARV, mmHg	-	0.303 (0.091 – 0.515)	0.005	0.125 (-0.070 – 0.319)	0.207
	VIM, mmHg	-	0.248 (0.034 – 0.461)	0.023	0.116 (-0.069 – 0.301)	0.218
	**Nighttime**					
	SD, mmHg	-	0.050 (-0.168 – 0.268)	0.651	0.107 (-0.077 – 0.291)	0.252
	CV, %	-	-0.062 (-0.280 – 0.155)	0.572	0.051 (-0.137 – 0.239)	0.594
	ARV, mmHg	-	0.065 (-0.152 – 0.281)	0.555	0.093 (-0.093 – 0.279)	0.323
		VCI	-0.183 (-0.477 – 0.111)	0.215	-0.047 (-0.331 – 0.236)	0.736
		COD	0.193 (-0.360 – 0.746)	0.483	0.174 (-0.346 – 0.695)	0.499
		HF	0.115 (-0.365 – 0.594)	0.633	0.142 (-0.244 – 0.529)	0.462
		Control	0.707 (0.238 – 1.176)	0.004	0.545 (0.131 – 0.958)	0.011
	VIM, mmHg	-	0.022 (-0.196 – 0.240)	0.840	0.094 (-0.090 – 0.278)	0.315

ARV: average real variability; CI: confidence interval; COD: carotid occlusive disease; CV: coefficient of variation; DBP: diastolic blood pressure; HF: heart failure; SBP: systolic blood pressure; SD: standard deviation; VCI: vascular cognitive impairment; VIM: variability independent of the mean; WMH: white matter hyperintensity.

[a] Adjusted for age, sex, mean 24-hour SBP or DBP (for analyses of SBP or DBP determinants respectively), use of blood pressure lowering medication and body mass index.

Supplementary Table 4. Sensitivity analyses: cross-sectional associations between blood pressure measurements and WMH volume (excluding participants using beta-blockers).

		Stratum	Crude standardized β (95% CI)	p-value	Adjusted standardized β (95% CI)[a]	p-value
Systolic blood pressure	**24-hour**					
	Mean, mmHg	-	0.439 (0.156 – 0.721)	0.003	0.391 (0.137 – 0.646)	0.003
	ARV, mmHg	-	0.253 (-0.020 – 0.527)	0.069	-0.011 (-0.262 – 0.239)	0.928
	Nocturnal dipping, ratio	-	0.278 (0.010 – 0.546)	0.043	0.189 (-0.044 – 0.421)	0.111
	**Daytime**					
	ARV, mmHg	-	0.261 (-0.002 – 0.523)	0.052	-0.035 (-0.276 – 0.205)	0.771
	**Nighttime**					
	ARV, mmHg	-	0.006 (-0.264 – 0.276)	0.966	-0.022 (-0.254 – 0.210)	0.851
Diastolic blood pressure	**24-hour**					
	Mean, mmHg	-	0.282 (-0.022 – 0.585)	0.069	0.543 (0.282 – 0.804)	< 0.001
	ARV, mmHg	-	0.375 (0.104 – 0.646)	0.007	0.153 (-0.092 – 0.397)	0.219
	Nocturnal dipping, ratio	-	0.268 (0.008 – 0.528)	0.044	0.223 (0.009 – 0.438)	0.042
	**Daytime**					
	ARV, mmHg	-	0.416 (0.151 – 0.681)	0.002	0.140 (-0.101 – 0.381)	0.251
	**Nighttime**					
	ARV, mmHg	-	0.035 (-0.215 – 0.285)	0.782	0.046 (-0.170 – 0.263)	0.672
		VCI	-0.173 (-0.496 – 0.150)	0.284	-0.024 (-0.332 – 0.284)	0.875
		COD	0.140 (-0.416 – 0.696)	0.610	0.146 (-0.375 – 0.667)	0.569
		HF	-	-	-	-
		Control	0.704 (0.195 – 1.214)	0.008	0.400 (-0.052 – 0.851)	0.081

Of the 177 participants included in the cross-sectional analyses, 63 participants used beta-blockers. This leaves 114 participants for the sensitivity analyses. We could not calculate the β's for the association between nighttime DBP ARV and WMH volume for the HF participants, because there were only 5 HF participants available for this analysis.

ARV: average real variability; CI: confidence interval; COD: carotid occlusive disease; DBP: diastolic blood pressure; HF: heart failure; SBP: systolic blood pressure; VCI: vascular cognitive impairment; WMH: white matter hyperintensity.

[a] Adjusted for age, sex, mean 24-hour SBP or DBP (for analyses of SBP or DBP determinants respectively), use of blood pressure lowering medication and body mass index.

Supplementary Table 5. Baseline characteristics of the participants by participants with or without follow-up.

Characteristic	Participants without follow-up N = 66	Participants with follow-up N = 91	p-value
Female	16 (24.2)	36 (39.6)	0.059
Age, years	67.0 ± 9.1	65.4 ± 7.7	0.245
**Participant group**			
Vascular cognitive impairment	13 (19.7)	19 (20.9)	1.000
Carotid occlusive disease	7 (10.6)	25 (27.5)	0.015
Heart failure	30 (45.5)	16 (17.6)	< 0.001
Control	16 (24.2)	31 (34.1)	0.218
**Vascular risk factors**			
Current smoking	9 (13.6)	18 (19.8)	0.393
Hypertension	47 (71.2)	61 (67.0)	0.605
Hyperlipidaemia	44 (66.7)	55 (60.4)	0.503
Diabetes mellitus	3 (4.5)	8 (8.8)	0.360
BMI, kg/m^2	26.1 ± 3.5	26.7 ± 3.6	0.258
History of cerebrovascular or cardiovascular disease	43 (65.2)	52 (57.1)	0.326
**Antihypertensive medication**			
Any medication	44 (66.7)	57 (62.6)	0.617
**Blood pressure measurements**			
SBP 24-h mean, mmHg	118.5 ± 13.0	125.0 ± 14.1	0.006
DBP 24-h mean, mmHg	71.6 ± 8.8	74.5 ± 9.3	0.080
SBP 24-h ARV, mmHg	9.4 ± 2.1	10.2 ± 2.7	0.080
DBP 24-h ARV, mmHg	7.5 ± 1.7	8.0 ± 2.0	0.202
SBP nocturnal dipping, ratio	0.90 ± 0.08	0.88 ± 0.08	0.148
DBP nocturnal dipping, ratio	0.86 ± 0.10	0.84 ± 0.11	0.107
**WMH volume**			
At baseline, ml	1.03 (0.39-8.72)	1.06 (0.38 – 3.11)	0.593
At baseline, % of ICV	0.07 (0.03-0.58)	0.08 (0.03 – 0.25)	0.646
At baseline, Fazekas grade 2 or 3	20 (33.3)	20 (22.2)	0.187
**Cerebral perfusion**			
At baseline, ml/min/100 g (N = 151)	57.2 ± 14.5	56.5 ± 14.8	0.865

Data are presented a N (%) for categorical variables and mean ± SD for continuous variables, except for WMH volume, which is presented as median (interquartile range). The patients which had their 24-hour ABPM during the follow-up after two years are not included in this comparison.
ARV: average real variability; BMI: body mass index; DBP: diastolic blood pressure; ICV: intracranial volume; SBP: systolic blood pressure; WMH: white matter hyperintensity.

Supplementary Table 6. Longitudinal associations between blood pressure measurements and progression of WMH volume (continuous).

		Stratum	Crude standardized β (95% CI)	p-value	Adjusted standardized β (95% CI)[a]	p-value
Systolic blood pressure	**24-hour**					
	Mean, mmHg	-	0.111 (-0.007 – 0.229)	0.064	0.135 (0.018 – 0.251)	0.024
	SD, mmHg	-	0.045 (-0.070 – 0.160)	0.436	-0.021 (-0.144 – 0.101)	0.729
	CV, %	-	-0.007 (-0.127 – 0.114)	0.912	-0.23 (-0.138 – 0.091)	0.685
	ARV, mmHg	-	0.092 (-0.013 – 0.196)	0.084	0.028 (-0.083 – 0.138)	0.617
	VIM, mmHg	-	0.006 (-0.113 – 0.125)	0.921	-0.023 (-0.137 – 0.091)	0.685
	Nocturnal dipping, ratio	-	0.020 (-0.104 – 0.143)	0.752	0.004 (-0.116 – 0.124)	0.949
	**Daytime**					
	SD, mmHg	-	0.055 (-0.058 – 0.169)	0.337	-0.011 (-0.128 – 0.106)	0.852
	CV, %	-	0.010 (-0.115 – 0.134)	0.878	-0.025 (-0.145 – 0.094)	0.673
	ARV, mmHg	-	0.092 (-0.009 – 0.193)	0.074	0.024 (-0.082 – 0.129)	0.657
	VIM, mmHg	-	0.032 (-0.087 – 0.151)	0.593	-0.019 (-0.135 – 0.097)	0.743
	**Nighttime**					
	SD, mmHg	-	0.046 (-0.054 – 0.145)	0.362	0.028 (-0.071 – 0.128)	0.574
	CV, %	-	0.023 (-0.081 – 0.127)	0.663	0.026 (-0.074 – 0.126)	0.608
	ARV, mmHg	-	0.027 (-0.079 – 0.134)	0.611	0.013 (-0.091 – 0.118)	0.802
		VCI	0.338 (0.119 – 0.557)	0.005	0.351 (0.069 – 0.634)	0.019
		COD	-0.185 (-0.381 – 0.012)	0.064	-0.198 (-0.401 – 0.005)	0.055
		HF	0.007 (-0.156 – 0.170)	0.925	0.002 (-0.219 – 0.224)	0.980
		Control	0.068 (-0.182 – 0.318)	0.583	-0.026 (-0.285 – 0.232)	0.835
	VIM, mmHg	-	0.033 (-0.069 – 0.134)	0.526	0.026 (-0.072 – 0.125)	0.596
Diastolic blood pressure	**24-hour**					
	Mean, mmHg	-	0.031 (-0.083 – 0.144)	0.592	0.092 (-0.024 – 0.209)	0.119
	SD, mmHg	-	0.075 (-0.037 – 0.187)	0.186	0.055 (-0.061 – 0.171)	0.347
	CV, %	-	0.053 (-0.060 – 0.166)	0.352	0.045 (-0.066 – 0.156)	0.423
	ARV, mmHg	-	0.170 (0.064 – 0.276)	0.002	0.144 (0.030 – 0.258)	0.014
	VIM, mmHg	-	0.064 (-0.048 – 0.177)	0.261	0.046 (-0.062 – 0.155)	0.399
	Nocturnal dipping, ratio	-	-0.063 (-0.183 – 0.057)	0.302	-0.046 (-0.164 – 0.071)	0.433
	**Daytime**					
	SD, mmHg	-	0.077 (-0.035 – 0.189)	0.177	0.044 (-0.068 – 0.156)	0.438
	CV, %	-	0.046 (-0.073 – 0.165)	0.441	0.027 (-0.091 – 0.144)	0.654
	ARV, mmHg	-	0.175 (0.069 – 0.281)	0.001	0.137 (0.026 – 0.248)	0.016

	VIM, mmHg	-	0.067 (-0.047 – 0.182)	0.247	0.037 (-0.075 – 0.148)	0.516
	**Nighttime**					
	SD, mmHg	-	0.033 (-0.072 – 0.138)	0.538	0.063 (-0.043 – 0.169)	0.242
	CV, %	-	0.030 (-0.082 – 0.142)	0.597	0.060 (-0.050 – 0.169)	0.282
	ARV, mmHg	-	0.029 (-0.122 – 0.180)	0.700	0.050 (-0.105 – 0.206)	0.522
	VIM, mmHg	-	0.033 (-0.074 – 0.139)	0.544	0.062 (-0.044 – 0.169)	0.246

The longitudinal analyses are based on the data of 91 patients. Median follow-up was 2.1 years (IQR 2.1-2.3 years).

ARV: average real variability; CI: confidence interval; COD: carotid occlusive disease; CV: coefficient of variation; DBP: diastolic blood pressure; HF: heart failure; SBP: systolic blood pressure; SD: standard deviation; VCI: vascular cognitive impairment; VIM: variability independent of the mean; WMH: white matter hyperintensity.

[a] Adjusted for age, sex, mean 24-hour SBP or DBP (for analyses of SBP or DBP determinants respectively), use of blood pressure lowering medication and body mass index.

Supplementary Table 7. Longitudinal associations between blood pressure measurements and progression of WMH volume (dichotomized).

		Stratum	Crude standardized OR (95% CI)	p-value	Adjusted standardized OR (95% CI)[a]	p-value
**Systolic blood pressure**	**24-hour**					
	Mean, mmHg	-	1.55 (0.98 – 2.45)	0.063	1.42 (0.87 – 2.34)	0.163
	SD, mmHg	-	1.09 (0.72 – 1.67)	0.679	0.82 (0.48 – 1.41)	0.472
	CV, %	-	0.90 (0.58 – 1.40)	0.627	0.83 (0.50 – 1.39)	0.478
	ARV, mmHg	-	1.14 (0.77 – 1.68)	0.508	0.79 (0.49 – 1.30)	0.357
	VIM, mmHg	-	0.94 (0.61 – 1.46)	0.786	0.83 (0.50 – 1.38)	0.470
	Nocturnal dipping, ratio	-	1.24 (0.79 – 1.93)	0.351	0.93 (0.56 – 1.56)	0.793
	**Daytime**					
	SD, mmHg	-	1.12 (0.74 – 1.69)	0.590	0.79 (0.48 – 1.31)	0.356
	CV, %	-	0.98 (0.63 – 1.54)	0.936	0.76 (0.45 – 1.27)	0.294
	ARV, mmHg	-	1.16 (0.80 – 1.68)	0.443	0.83 (0.53 – 1.31)	0.429
	VIM, mmHg	-	1.05 (0.68 – 1.61)	0.829	0.77 (0.47 – 1.28)	0.314
	**Nighttime**					
	SD, mmHg	-	1.04 (0.72 – 1.50)	0.829	0.90 (0.59 – 1.37)	0.609
	CV, %	-	0.94 (0.64 – 1.38)	0.755	0.90 (0.59 – 1.37)	0.616
	ARV, mmHg	-	1.05 (0.71 – 1.55)	0.820	0.86 (0.56 – 1.34)	0.511
	VIM, mmHg	-	0.98 (0.68 – 1.43)	0.930	0.90 (0.59 – 1.36)	0.614
**Diastolic blood pressure**	**24-hour**					
	Mean, mmHg	-	1.09 (0.72 – 1.65)	0.686	1.18 (0.74 – 1.89)	0.482
	SD, mmHg	-	1.20 (0.79 – 1.81)	0.400	1.10 (0.66 – 1.82)	0.722
	CV, %	-	1.13 (0.74 – 1.72)	0.566	1.07 (0.66 – 1.73)	0.793
	ARV, mmHg	-	1.64 (1.07 – 2.52)	0.024	1.40 (0.83 – 2.36)	0.212
	VIM, mmHg	-	1.16 (0.77 – 1.77)	0.480	1.07 (0.67 – 1.72)	0.779
	Nocturnal dipping, ratio	-	1.12 (0.73 – 1.74)	0.600	1.02 (0.62 – 1.65)	0.953
	**Daytime**					
	SD, mmHg	-	1.42 (0.93 – 2.18)	0.109	1.25 (0.77 – 2.04)	0.366
	CV, %	-	1.41 (0.89 – 2.22)	0.141	1.23 (0.74 – 2.03)	0.432
	ARV, mmHg	-	1.73 (1.11 – 2.70)	0.016	1.55 (0.92 – 2.60)	0.101
	VIM, mmHg	-	1.43 (0.92 – 2.21)	0.113	1.24 (0.76 – 2.01)	0.394
	**Nighttime**					
	SD, mmHg	-	1.35 (0.91 – 2.01)	0.136	1.36 (0.85 – 2.17)	0.202
	CV, %	-	1.26 (0.83 – 1.92)	0.271	1.26 (0.78 – 2.04)	0.339

	ARV, mmHg	-	1.50 (0.86 – 2.62)	0.149	1.20 (0.63 – 2.30)	0.579
	VIM, mmHg	-	1.34 (0.90 – 2.01)	0.152	1.34 (0.84 – 2.14)	0.222

The longitudinal analyses are based on the data of 91 patients. Median follow-up was 2.1 years (IQR 2.1-2.3 years).
ARV: average real variability; CI: confidence interval; CV: coefficient of variation; DBP: diastolic blood pressure; OR: odds ratio; SBP: systolic blood pressure; SD: standard deviation; VIM: variability independent of the mean; WMH: white matter hyperintensity.
[a] Adjusted for age, sex, mean 24-hour SBP or DBP (for analyses of SBP or DBP determinants respectively), use of blood pressure lowering medication and body mass index.

Supplementary Table 8. Sensitivity analyses: longitudinal associations between blood pressure measurements and progression of WMH volume (continuous) (excluding participants using beta-blockers).

		Stratum	Crude standardized β (95% CI)	p-value	Adjusted standardized β (95% CI)[a]	p-value
**Systolic blood pressure**	**24-hour**					
	Mean, mmHg	-	0.111 (-0.044 – 0.266)	0.158	0.127 (-0.023 – 0.277)	0.097
	ARV, mmHg	-	0.103 (-0.031 – 0.236)	0.129	0.040 (-0.100 – 0.179)	0.571
	Nocturnal dipping, ratio	-	-0.003 (-0.156 – 0.149)	0.965	-0.002 (-0.148 – 0.145)	0.980
	**Daytime**					
	ARV, mmHg	-	0.103 (-0.020 – 0.226)	0.099	0.025 (-0.103 – 0.152)	0.700
	**Nighttime**					
	ARV, mmHg	-	0.004 (-0.129 – 0.137)	0.953	-0.006 (-0.134 – 0.123)	0.929
**Diastolic blood pressure**	**24-hour**					
	Mean, mmHg	-	-0.001 (-0.156 – 0.155)	0.991	0.095 (-0.060 – 0.251)	0.224
	ARV, mmHg	-	0.223 (0.090 – 0.356)	0.001	0.201 (0.066 – 0.336)	0.004
	Nocturnal dipping, ratio	-	-0.094 (-0.236 – 0.048)	0.191	-0.059 (-0.196 – 0.079)	0.397
	**Daytime**					
	ARV, mmHg	-	0.205 (0.077 – 0.333)	0.002	0.151 (0.020 – 0.282)	0.024
	**Nighttime**					
	ARV, mmHg	-	0.065 (-0.121 – 0.251)	0.490	0.101 (-0.088 – 0.289)	0.290

Of the 91 participants included in the cross-sectional analyses, 22 participants used beta-blockers. This leaves 69 participants for the sensitivity analyses.

ARV: average real variability; CI: confidence interval; DBP: diastolic blood pressure; SBP: systolic blood pressure; WMH: white matter hyperintensity.

[a] Adjusted for age, sex, mean 24-hour SBP or DBP (for analyses of SBP or DBP determinants respectively), use of blood pressure lowering medication and body mass index.

Supplementary Table 9. Mediation analyses to determine if cerebral perfusion acts as a mediator between BPV or nocturnal dipping and progression of WMH volume.

	**Direct effect of BPV or nocturnal dipping**		**Indirect effect of BPV or nocturnal dipping via cerebral perfusion**	
	**Adjusted unstandardized β (95% CI)[a]**	**p-value**	**Adjusted unstandardized β (95% CI)[a]**	**p-value**
**SBP**				
ARV 24-h, mmHg	0.017 (-0.030 – 0.063)	0.481	-0.001 (-0.011 – 0.008)	0.849
ARV day, mmHg	0.011 (-0.024 – 0.045)	0.543	-0.000 (-0.005 – 0.005)	0.967
ARV night, mmHg	0.004 (-0.018 – 0.025)	0.734	-0.000 (-0.004 – 0.003)	0.952
Nocturnal dipping, ratio	0.056 (-1.380 – 1.493)	0.938	0.029 (-0.333 – 0.445)	0.883
**DBP**				
ARV 24-h, mmHg	0.080 (0.015 – 0.146)	0.017	-0.001 (-0.018 – 0.015)	0.944
ARV day, mmHg	0.061 (0.011 – 0.111)	0.018	-0.001 (-0.017 – 0.012)	0.907
ARV night, mmHg	0.011 (-0.028 – 0.051)	0.570	-0.001 (-0.009 – 0.005)	0.885
Nocturnal dipping, ratio	-0.380 (-1.494 – 0.734)	0.499	-0.015 (-0.253 – 0.173)	0.885

ARV: average real variability; BPV: blood pressure variability; CI: confidence interval; DBP: diastolic blood pressure; SBP: systolic blood pressure; WMH: white matter hyperintensity.

[a] Adjusted for age, sex, mean 24-hour SBP or DBP (for analyses of SBP or DBP determinants respectively), use of blood pressure lowering medication and body mass index.

Supplementary Table 10. Effect moderation analyses to determine if cerebral perfusion acts as an effect moderator between BPV or nocturnal dipping and progression of WMH volume.

	**Stratification for cerebral perfusion**	**Adjusted standardized β (95% CI)[a]**	**p-value for regression coefficient**	**p-value for interaction**
**SBP**				
ARV 24-h, mmHg	Low	0.036 (-0.153 – 0.226)	0.694	0.451
	Middle	0.095 (-0.168 – 0.358)	0.463	
	High	0.061 (-0.147 – 0.270)	0.547	
ARV day, mmHg	Low	0.083 (-0.149 – 0.316)	0.465	0.220
	Middle	0.117 (-0.118 – 0.351)	0.313	
	High	-0.063 (-0.234 – 0.108)	0.455	
ARV night, mmHg	Low	-0.021 (-0.156 – 0.114)	0.749	0.142
	Middle	-0.061 (-0.344 – 0.222)	0.659	
	High	0.298 (0.141 – 0.455)	0.001	
Nocturnal dipping, ratio	Low	0.120 (-0.195 – 0.434)	0.437	0.943
	Middle	-0.019 (-0.272 – 0.233)	0.875	
	High	0.064 (-0.123 – 0.250)	0.485	
**DBP**				
ARV 24-h, mmHg	Low	0.210 (0.035 – 0.385)	0.021	0.042
	Middle	0.156 (-0.060 – 0.373)	0.149	
	High	0.052 (-0.155 – 0.259)	0.606	
ARV day, mmHg	Low	0.209 (0.037 – 0.381)	0.020	0.027
	Middle	0.150 (-0.047 – 0.346)	0.128	
	High	0.003 (-0.249 – 0.254)	0.983	
ARV night, mmHg	Low	-0.077 (-0.357 – 0.202)	0.571	0.353
	Middle	0.044 (-0.248 – 0.335)	0.758	
	High	0.159 (-0.065 – 0.384)	0.155	
Nocturnal dipping, ratio	Low	-0.026 (-0.264 – 0.213)	0.826	0.650
	Middle	-0.115 (-0.352 – 0.122)	0.325	
	High	0.012 (-0.188 – 0.211)	0.903	

ARV: average real variability; BPV: blood pressure variability; CI: confidence interval; DBP: diastolic blood pressure; SBP: systolic blood pressure; WMH: white matter hyperintensity.

[a] Adjusted for age, sex, mean 24-hour SBP or DBP (for analyses of SBP or DBP determinants respectively), use of blood pressure lowering medication and body mass index.
